# Intelligent breast cancer diagnosis with two-stage using mammogram images

**DOI:** 10.1038/s41598-024-65926-0

**Published:** 2024-07-19

**Authors:** Muhammad Yaqub, Feng Jinchao, Nazish Aijaz, Shahzad Ahmed, Atif Mehmood, Hao Jiang, Lan He

**Affiliations:** 1 School of Biomedical Sciences, Hunan University, Changsha, People’s Republic of China; 2 Faculty of Information Technology, Beijing University of Technology, Beijing, People’s Republic of China; 3 Department of Computer Science and Technology, Zhejiang Normal University, Jinhua, 321002 People’s Republic of China; 4 Department of Biomedical Informatics School of Life Sciences, Central South University, Changsha, 410013 Hunan People’s Republic of China

**Keywords:** Breast cancer, Mammograms, Atrous convolution-based attentive and adaptive Trans-Res-UNet, Modified mussel length-based eurasian oystercatcher optimization, Atrous convolution based attentive and adaptive multi-scale DenseNet, Breast cancer, Computer science, Medical imaging

## Abstract

Breast cancer (BC) significantly contributes to cancer-related mortality in women, underscoring the criticality of early detection for optimal patient outcomes. Mammography is a key tool for identifying and diagnosing breast abnormalities; however, accurately distinguishing malignant mass lesions remains challenging. To address this issue, we propose a novel deep learning approach for BC screening utilizing mammography images. Our proposed model comprises three distinct stages: data collection from established benchmark sources, image segmentation employing an Atrous Convolution-based Attentive and Adaptive Trans-Res-UNet (ACA-ATRUNet) architecture, and BC identification via an Atrous Convolution-based Attentive and Adaptive Multi-scale DenseNet (ACA-AMDN) model. The hyperparameters within the ACA-ATRUNet and ACA-AMDN models are optimized using the Modified Mussel Length-based Eurasian Oystercatcher Optimization (MML-EOO) algorithm. The performance is evaluated using a variety of metrics, and a comparative analysis against conventional methods is presented. Our experimental results reveal that the proposed BC detection framework attains superior precision rates in early disease detection, demonstrating its potential to enhance mammography-based screening methodologies.

## Introduction

Breast cancer (BC) is the most prevalent type of malignancy in women and is the second leading cause of cancer-related mortality among women^1^. One in every 36 female deaths is related to BC, or around 3% of all female deaths are caused by BC. In order to improve the survival rate of the patient, early BC identification is crucial^2^. Due to the huge lethality and high cost of cancer-related treatments, researchers are introducing increasingly accurate BC diagnostic models into practice^3,4^. Radiotherapists use mammography as an efficient imaging method to detect and screen the presence of BC. Mammography is the primary clinical test for BC and is quite accurate in predicting BC. Breast Mammography usually detects 80–90% of BC cases in females with no symptoms^5^. Identifying early signs of breast cancer, such as breast lumps and calcifications, is crucial for timely diagnosis and is effectively facilitated by mammography^6^.

Early detection of BC through mammography can greatly reduce the cost of treating BC. Although mammography is advantageous, screening BC from it consumes more time and requires expert knowledge for accurate detection. Also, the decision made by radiologists varies. Computer Assisted Diagnosis (CAD) software is created to help radiotherapists improve prediction accuracy while screening through mammography^7^. However, CAD software has a substantial risk of producing false positive and false negative outcomes^8^. As a result, it is crucial to improve doctors' detection effectiveness using CAD systems with deep learning approaches. Because of their diverse size, shape, and position, the BC cells are difficult to detect and localize automatically from the BC mammography images^9^. Several methods have been developed for fragmenting medical mammography images. Due to the limitations of each approach, segmentation of mammography images is proven to be a challenging task^10^. The recognition of signs of BC from breast mammography images is also a significant challenge. An essential stage in the analysis of mammography images is the feature extraction mechanism. Traditional approaches use handcrafted features to describe the image's content^11^. The neural network is developed as a replacement for automatically detecting the best characteristics. Additionally, incorrectly interpreting these images results in a dangerously inaccurate diagnosis^12^. Consider a false negative diagnosis, where a BC in its early stages is mistaken for a typical instance. One of the traditional methods for reducing these kinds of computational errors is feature selection (FS), which eliminates duplicate features and chooses a group of distinctive features. Distinctive features might not be granted the significance they should require in the classification process due to these duplicate features^13^.

To aid in decision-making, experts have used a variety of machine learning^14^ approaches in medical image interpretation over the last few years. However, the system's performance suffers concerning efficacy and precision because of the complicated nature of traditional machine learning methods, like segmentation, feature extraction, preprocessing, and others^15,16^. The newly developed deep learning techniques tackle conventional machine learning problems. This technique can successfully represent features to perform the tasks of object localization and image classification^17^. Medical professionals can use their expertise to connect dataset features to facts, which is difficult for machine learning techniques to accomplish. In contrast to conventional techniques that rely on manual methods, deep learning eliminates this problem by including processing and feature engineering as a component of learning^18^. CNN is the most widely used deep learning method for image processing. The 2D input-image configuration can specifically alter the CNN design. However, it achieved a precision of only 88%, which has to be further raised to be more effective than the state-of-the-art methods^19^. Hence, this paper generates and implements a novel deep-learning approach for early screening and BC.

The main achievements that have been performed in this work are listed below.

- Proposed a two-step BC detection method improving efficiency and precision in mammogram segmentation and diagnosis.
- Developed MLL-EOO module to optimize feature extraction in Trans-Rs-UNet and Multi-scale DenseNet, enhancing segmentation.
- Utilized heuristic-assisted Trans-Res-UNet and Multi-scale DenseNet for intelligent BC detection.
- Implemented Modified Mussel Length-based Eurasian Oystercatcher Optimization algorithm for fine-tuning the deep learning models.
- The proposed MLL-EOO module improves the limitation of feature region size selection.

The further sections in this paper are given below. Section "Motivation" carries the literature review done in examining the pre-existing works. Section "Methods" contains the development of the intelligent BC detection model with heuristic-assisted Trans-Res-UNet and Multi-scale DenseNet using mammogram images; provides a detailed view of the Atrous Convolution-based Attentive and Adaptive BC segmentation model using mammogram images; comprises the architectural representation of the Atrous Convolution-based Attentive and Adaptive BC detection model using mammogram images. Section "Results and discussion" gives the experimental outputs and the discussions that are carried out regarding the generated results. Section 5 summarizes the developed deep learning-based BC detection framework.

## Motivation

### Literature review

Das et al. (2021) proposed a stacked ensemble model for breast cancer (BC) classification that combined breast histopathology images and gene expression data^20^. The model incorporated the Convex Hull Algorithm and t-Distributed Stochastic Neighbor embedding to transform the 1D gene expression data into images. The dataset and its decomposed forms were utilized to enhance performance. Three convolutional neural networks (CNNs) served as the foundational classifiers in the first stage, with the data decomposed using Variational Mode Decomposition and Empirical Wavelet Transform. The results of the first stage were used to train the second-stage classifier. The model employed gene expression data from Mendeley to create 2D datasets. Training and validation were conducted using synthetic and photographic datasets of breast histopathology. The proposed method demonstrated improved performance, highlighting its effectiveness in BC classification.

In 2021, Saber et al.^21^ proposed a Transfer Learning (TL) method using ResNet50, Inception V3, VGG-16, ResNet, and VGG-19 networks for feature extraction from the MIAS dataset to aid in the automatic identification and classification of breast cancer (BC) susceptible areas. TL of the VGG16 model demonstrated effective categorization of mammogram breast images for BC diagnosis. In 2022, Jiang et al.^22^ introduced the Probabilistic Anchor Assignment (PAA) technique to accurately identify and classify mammograms as malignant or benign, improving prognosis ability. The proposed framework included a single-stage PAA-based detector to identify abnormal tumor areas and a two-branch ROI detector for tumor categorization, incorporating a Threshold-adaptive Post-processing Algorithm for complex breast data. The model was trained and evaluated using publicly available mammogram databases, demonstrating enhanced classification accuracy compared to existing techniques.

In 2022, Kavitha et al.^23^ presented a novel computerized mammogram-based breast cancer (BC) diagnosis framework. The framework employed median filtering for preprocessing to eliminate irrelevant data in mammographic images. BC segmentation was achieved using the Optimal Kapur's Multilevel Thresholding with Shell Game Optimization (OKMT-SGO) method. The proposed model incorporated a Backpropagation Neural Network (BPNN) classifier and a CapsNet feature extractor for BC identification. Evaluation using benchmark DDSM and Mini-MIAS datasets showcased the superior performance of the proposed method in diagnostic accuracy. In 2022, Kumari and Jagadesh^24^ employed feature selection techniques to enhance classifier performance. Intensity, texture, and shape-based features were extracted from preprocessed medical images. The selected features were used with the XGBoost classifier and compared to other classifiers. The MIAS database was utilized for experimentation and evaluation. Results showed that the proposed XGBoost framework outperformed other feature selection techniques in categorising MIAS mammography images as abnormal or normal, demonstrating its superior performance.

In 2021, Patil and Biradar^25^ proposed an improved hybrid classification model for mammogram-based breast cancer (BC) detection. The approach involved image preprocessing, feature extraction, segmentation^26,27^, and identification stages. A median filter was used for noise removal, and the Firefly updated Chicken-based Optimization (FC-CSO) algorithm was employed for tumor segmentation. Features were extracted and fed into a Recurrent Neural Network (RNN) and a Convolutional Neural Network (CNN) for classification^28^. The combination of the two models achieved superior accuracy compared to traditional classifiers.

In 2022, Pramanik et al.^29^ proposed a breast mass categorization system using mammograms. The VGG16 architecture with an attention mechanism extracted deep features from mammography images. The Social Ski-Driver (SSD) technique and Adaptive Beta Hill Climbing search strategy were employed to obtain optimal features. The K-Nearest Neighbors (KNN) classifier utilized these features for data classification. The suggested model demonstrated successful recognition and discrimination between healthy and cancerous breasts. Remarkably, the framework achieved higher precision by utilizing only 25% of the attention-aided VGG16 model's features on a publicly available dataset. In 2020, Zheng et al.^30^ introduced the DL-Assisted Efficient AdaBoost Algorithm (DLA-EABA) for breast cancer (BC) identification. The study investigated the characterization of breast masses using transfer learning from diverse imaging modalities, such as mammography, MRI, digital breast tomosynthesis, and ultrasound. The deep learning model incorporated LSTM layers, convolutional layers, fully connected layers, max-pooling layers, activation layers, and error estimation for classification. The fusion of machine learning approaches with feature selection and extraction methods was examined, and the model's performance was evaluated against existing segmentation methods and conventional classifiers.

### Problem statement and objectives

Breast cancer screening through mammography is crucial in early detection, offering the potential for more successful treatment outcomes. However, challenges arise in accurately interpreting mammograms, particularly in women with dense breasts, leading to increased rates of false predictions. Furthermore, normal mammogram results do not guarantee the absence of breast cancer, underscoring the limitations of relying solely on this screening method. In addition, false diagnoses can subject women to unnecessary radiation exposure. The complexity of handling the large volume of mammography images and the variability in prediction outcomes among radiologists further highlight the limitations of traditional breast cancer detection approaches. To address these challenges and improve the accuracy of breast cancer prediction while minimizing errors, we propose adopting deep learning techniques for breast cancer detection from mammography images. This novel approach aims to enhance the precision of predictions and mitigate the generation of false errors, offering a more efficient and reliable method for breast cancer detection.

Table 1 presents a comprehensive overview of current breast cancer (BC) detection techniques, along with their respective merits and demerits^20–25,29,30^. CNN and Empirical Wavelet Transform (EWT) demonstrate improved accuracy, recall, and precision detection rates but introduce hardware and time complexity. VGG achieves higher accuracy, AUC, and sensitivity, enhancing system robustness primarily for prognosis objectives. PAA extracts peripheral regions to identify diseases but incurs computational burdens that degrade system robustness. BPNN offers discriminative features for detection but lacks parameter tuning for further enhancement. XGBoost selects notable features for increased detection accuracy but does not support large-scale dimensional datasets. CNN significantly extracts boundary-level regions for accurate results yet, blur or noise in images degrades system robustness and can lead to misdiagnosis. KNN acquires deep and optimal features for cancer region detection but suffers from high time complexity and premature convergence. CNN-LSTM obtains desirable early-stage disease detection but is susceptible to overfitting. These challenges underscore the need to develop and implement an accurate BC detection approach using deep learning techniques.

**Table 1:** Merits and demerits of traditional BC detection methods.

Author [citation]	Methodology	Features	Challenges
Das et al.^20^	CNN and EWT	It improves the detection rate regarding the accuracy, recall, precision, etc	It causes hardware complexity as well as time complexity
Saber et al*.*^21^	VGG	It achieves more accuracy, AUC, and sensitivity, enhancing the system's robustness	It is further developing for prognosis objectives
Jiang et al*.*^22^	PAA	It extracts the peripheral regions to get the features for identifying the diseases	It causes a computational burden that degrades the robustness of the system
Kavitha et al*.*^23^	BPNN	It offers discriminative features for reaching a higher detection rate	It does not tune the parameters used in the model for further enhancement
Kumari and Jagadesh^24^	XGBoost	It chooses the noteworthy features for increasing the detection accuracy	It does not support large-scale dimensional datasets
Patil and Biradar^25^	CNN	It significantly extracts the boundary-level regions for estimating the appropriate results	The blur or noise present in images degrades the system's robustness and misdiagnoses the disease
Pramanik et al*.*^29^	KNN	It acquires deep and optimal features for detecting the cancer regions in images	Time complexity and premature convergence rate occur
Zheng et al*.*^30^	CNN-LSTM	It obtains the desired value to detect the disease at its early stages	It causes the overfitting problem

## Methods

### Intelligent breast cancer detection model with heuristic-assisted Trans-Res-U-Net and multi-scale DenseNet using mammogram images

#### Proposed model and description

Traditional breast cancer detection methods like mammography, MRI, and CAD tools frequently fail to diagnose early stages, especially in women with dense breast tissues or surgical histories, leading to misdiagnoses and unnecessary treatments. These techniques, while common, often result in inaccurate predictions and can expose healthy individuals to harmful radiation. Despite the development of advanced imaging technologies such as PET and Molecular Breast Imaging, their high costs limit accessibility primarily to high-risk patients. To improve accuracy and reduce costs, a new deep learning-based framework for breast cancer detection has been introduced.

In the developed deep learning-based BC detection framework, the mammogram images are primarily obtained from standardized mammogram image data sources. These raw images are then provided to the developed ACA-ATRUNet classifier for the segmentation process. Before classification, these images are segmented to improve the overall accuracy of the further detection process. The segmented images are now given to the implemented ACA-AMDN framework for classifying the BC images. An enhanced metaheuristic optimization algorithm called the MML-EOO algorithm is suggested to reduce the processing complexity and computational time. The recommended MML-EOO algorithm optimizes the hidden neurons in the ACA-ATRUNet classifier, Epochs in the ACA-ATRUNet classifier, steps per epochs in the ACA-ATRUNet classifier, hidden neurons in the ACA-AMDN classifier, epochs in the ACA-AMDN classifier, and the batch size in the ACA-AMDN classifier, respectively. These optimized parameters help in fastening the entire detection process. The final detection image of BC is obtained from the ACA-AMDN classifier.

### Atrous convolution-based attentive and adaptive breast cancer segmentation model using mammogram images

#### ACA-ATRUNet-based breast cancer segmentation

The obtained raw mammogram images $B{C}_{fs}^{img}$ are given to the generated ACA-ATRUNet for image segmentation where $img$ symbolizes images, $fs$ implies feature selection. ACA-ATRUNet is developed by replacing the normal convolutional layer in the Trans-Res-UNet with an Atrous convolutional layer and including an attention mechanism. The entire process is repeated several numbers of times (Multi-scale) before producing the final segmented BC image output $S{I}_{ad}^{TRU}$. With the aid of the recommended MML-EOO algorithm, the elements in the ACA-ATRUNet structure are adjusted to increase the precision of the segmented BC images. The elements, such as epochs, steps per epochs, and the hidden neurons in the ACA-ATRUNet, are optimized by the suggested MML-EOO algorithm. This optimization is done to reluimize the dice coefficient and the accuracy between the mask images and the segmented BC images. The main idea behind this parameter optimization is mathematically represented in Eq. (1).

1 $$ S1 = \mathop {\arg \,\min }\limits_{{\left\{ {hi_{lm}^{TRU} ,eh_{kl}^{TRU} ,se_{jk}^{TRU} } \right\}}} \,\left( {\frac{1}{Dice + Arcy}} \right) $$

The term $S1$ in Eq. (1) denotes the objective function of the developed ACA-ATRUNet, $h{i}_{lm}^{TRU}$ denotes the optimally adjusted number of hidden neurons, $e{h}_{kl}^{TRU}$ denotes the optimally adjusted number of epochs, $s{e}_{jk}^{TRU}$ denotes the number of optimally adjusted steps per epoch, $Dice$ signifies the dice co-efficient between the mask image and the segmented BC image, and $Arcy$ represents the accuracy. The steps per epoch are tuned in the range $\left[\text{300,1000}\right]$, the hidden neurons are tuned in the range $\left[\text{5,255}\right]$, and the epochs are tuned in the range $\left[\text{5,50}\right]$. These parameters are tuned to maximize the Dice coefficient and accuracy. The Dice coefficient is the overlap among the masked and segmented images. The dice coefficient between the mask image and the segmented image is given by Eq. (2).

2 $$Dice\left(M{L}_{ma}^{am},S{I}_{ad}^{TRU}\right)=\frac{2\left(M{I}_{ma}^{am}\cap S{I}_{ad}^{TRU}\right)}{M{I}_{ma}^{am}+S{I}_{ad}^{TRU}}$$

The term $M{I}_{ma}^{am}$ in Eq. (2) denotes the mask images and $S{I}_{ad}^{TRU}$ represents the segmented BC image. The accuracy $Arcy$ evaluated using Eq. (3).

3 $$\text{Arcy} = \frac{TU+VW}{TU+TV+{\text{VW}}+VX}$$

**Figure 1:**
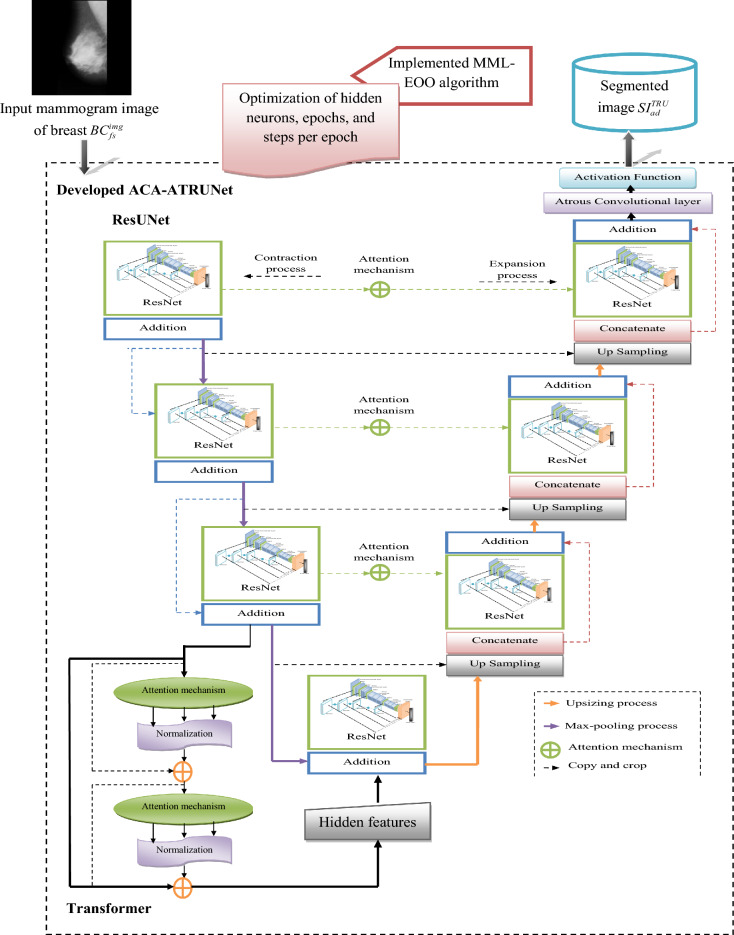
Pictorial representation of implemented ACA-ATRUNet-based BC mammogram image segmentation.

In Eq. (6), the terms $VW$ represent the true negative, $TU$ represents the true positive, $VX$ represents the false negative, and $TV$ represents the false positive, respectively. The pictorial representation of the implemented ACA-ATRUNEt-based BC mammogram image segmentation is provided in Fig. 1.

### Architectural representation of atrous convolution-based attentive and adaptive breast cancer detection model using mammogram images

#### ACA-AMDN-based breast cancer detection

The segmented images from the ACA-ATRUNet $S{I}_{ad}^{TRU}$ are fed to the developed ACA-AMDN structure for BC image classification. ACA-AMDN is developed by replacing the normal convolutional layer in the DenseNet with an Atrous convolutional layer and including an attention mechanism. The process is repeated several times (Multi-scale) in the DenseNet structure before producing the final classification output. The classified image output of BC mammogram images is given by $C{I}_{hs}^{MDN}$. The parameters are optimized in the ACA-AMDN structure with the assistance of the implemented MML-EOO algorithm. The parameters like epochs, batch size, and the hidden neurons in the Multi-scale DenseNet are optimally tuned with the help of the proposed MML-EOO algorithm. This optimization aims at maximizing accuracy and minimizing False Positive Rate (FPR). The major contribution behind this parameter optimization is formulated as in Eq. (4)

4 $$ S2 = \mathop {\arg \,\min }\limits_{{\left\{ {hi_{ml}^{MDN} ,eh_{lk}^{MDN} ,bs_{kj}^{MDN} } \right\}}} \,\left( {\frac{1}{Arcy} + XY} \right) $$

The term $S2$ in Eq. (4) denotes the objective function of the developed ACA-AMDN, $h{i}_{ml}^{MDN}$ denotes the optimally adjusted number of hidden neurons, $e{h}_{lk}^{MDN}$ denotes the optimally adjusted number of epochs, $b{s}_{kj}^{MGN}$ denotes the number of optimally adjusted steps per epoch, and $XY$ denotes the FPR. The batch size is tuned as $\left[2, 4, 8, 16, 32, 64\right]$ the hidden neurons are tuned in the range $\left[5, 255\right]$, and the epochs are tuned in the range $\left[5, 50\right]$. These parameters are optimized to maximize the accuracy and minimize the FPR. The FPR is computed using Eq. (5) as follows.

5 $$XY=\frac{\text{TV}}{VX+TV}$$

**Figure 2:**
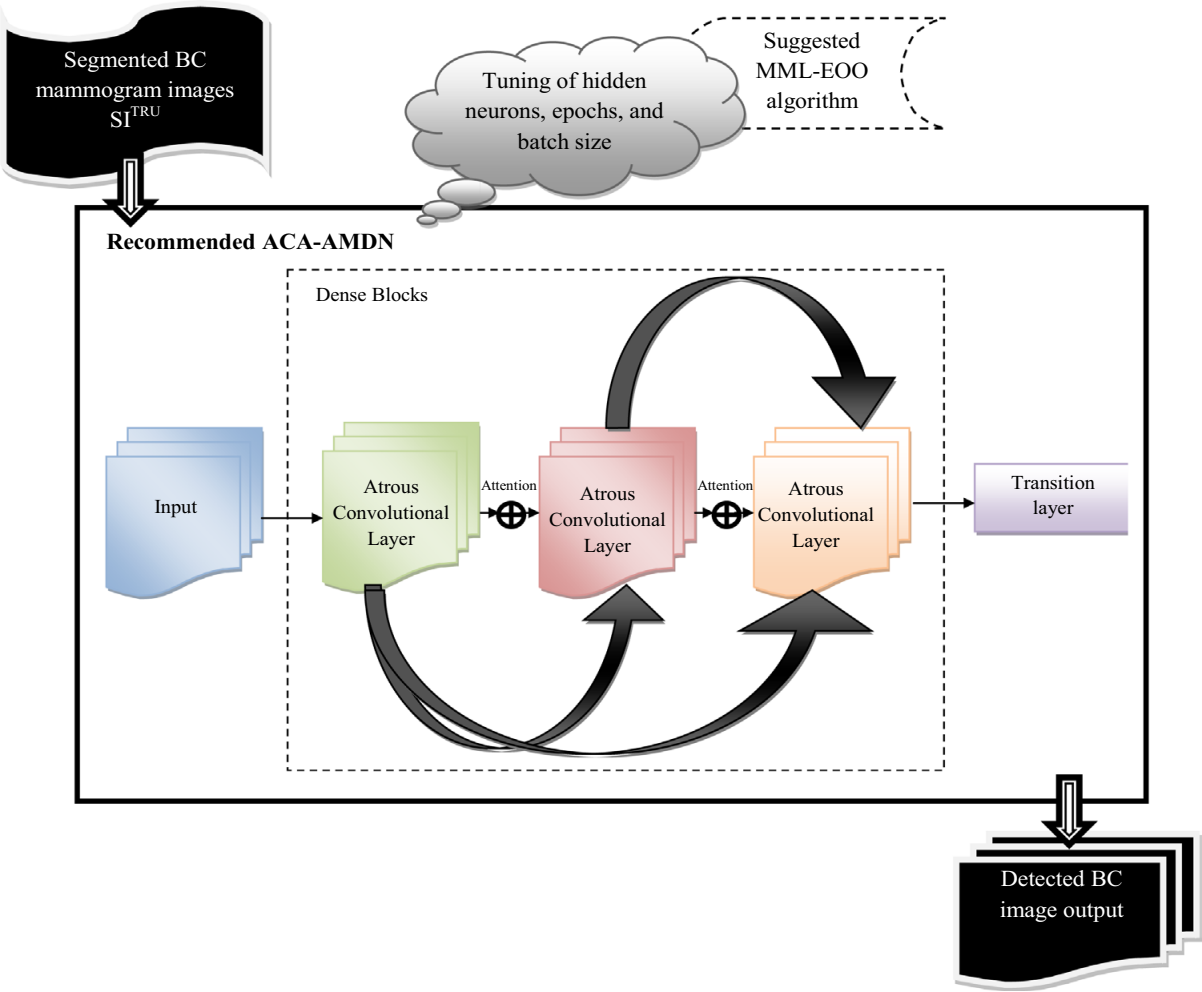
A pictorial illustration of the implemented ACA-AMDN-based BC classification model.

The pictorial illustration of the implemented ACA-AMDN-based BC classification is shown in Fig. 2.

#### Proposed MML-EOO

By optimizing the epoch, hidden neurons, step size of Trans-Rs-UNet and epoch, hidden neurons, and batch size of Multi-scale DenseNet, the final prediction result of the generated BC classification model can be improved. The suggested MML-EOO algorithm achieves this parameter optimization. The suggested MML-EOO algorithm achieves this parameter optimization. Because of its balanced exploitation and exploration and the capacity to eliminate local optimums, the EOO^31^ algorithm is used in this paper. Due to the oyster size selection constraint, however, this technique cannot resolve challenging real-time issues. As a result, the EOO algorithm's oyster size constraint $O$ is upgraded using the formula provided in Eq. (6).

6 $$O=5-r*\left(\frac{2}{R}\right)$$

The term $r$ in Eq. (10) represents the current iteration value, $R$ symbolizes the maximum iteration count, and $O$ denotes the size of the oyster. The value of $O$ is in the range $\left[\text{3,5}\right]$ in the traditional EOO algorithm, which is upgraded using Eq. (10) in the developed MML-EOO algorithm. The value $O$ decreases linearly from 50 to 30 mm in the suggested MML-EOO algorithm. The value $O$ in Eq. (6) is used to update the size of the oyster in Eqs. 7, 8, 9, and 10. The exploration of the EO is described as follows. The amount of energy that is available in the EO $K$ at the final stage of hunting the oyster is given by Eq. (7).

7 $$K=J+N+O*f*\left({P}_{m}-{P}_{r-1}\right)$$

This size of the oyster $O$ in Eq. (7) is upgraded using the fitness-based concept provided in Eq. (6). In Eq. (11),$N$ denotes the current energy requirement,$J$ denotes the time requirement of the EO to open the ideal oyster, and $f$ represents a number in random in the range $\left[\text{0,1}\right]$ that is selected to increase the predictability in the search area. The value of the available energy in the EO $K$ varies inversely as the iteration count $r$. The position in which the ideal oyster is found available is provided in Eq. (8).

8 $${P}_{r}={P}_{r-1}*Q$$

The term $Q$ in Eq. (8) represents the amount of energy the EO obtained from eating the ideal oyster of size $O$ and ${P}_{r}$ represents the position of the ideal oyster. The value of $J$ and the value of $Q$ relies on $O$. The time required to open a selected oyster $J$ is formulated as in Eq. (5).

9 $$J=\left(\left(\frac{O-3}{5-3}\right)*10\right)-5$$

The value of $O$ in Eq. (9) is updated using the fitness-based concept provided in Eq. (10). The presently available energy in the bird is computed as in Eq. (10).

10 $$D=\left(\frac{r-1}{s-1}\right)-0.5;r>1$$

The calorie that can be obtained by consuming the oyster $Q$ is given in Eq. (11).

11 $$Q=\left(\left(\frac{O-3}{5-3}\right)*2\right)+0.6$$

The value of $Q$ in Eq. (11) is updated using Eq. (6). If the time is negative, it represents that the bird has reached its maximum capacity in opening the oyster and cannot further spend energy in opening it. This is considered an exceptional case.$N$ remains constant in the last iteration and its preceding iteration. Thus $N$ and $J$ will have a negative value. The main contribution of the EOO algorithm is given as follows.

1. The precision of selecting a mussel by calculating the time needed to break one is calculated using the bird's energy and the mussel's size as variables to estimate the anticipated location of the desired food.
2. The random numbers entered during optimization help investigate new areas during each cycle. Avoid a local minimum issue as a result.
3. The random numbers used at each optimization stage ensure research and application.

**Figure:**
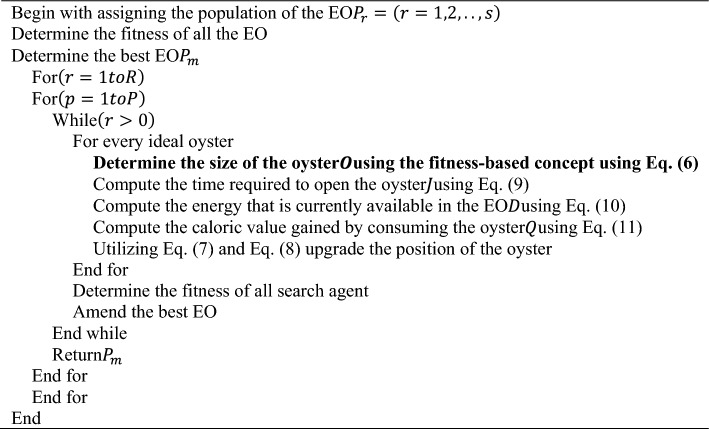
Algorithm 1: Proposed MML-EOO pseudocode

The flowchart of the suggested MML-EOO algorithm is given in Appendix (A) supplementary information. The pseudocode of the proposed MML-EOO algorithm is presented in Algorithm 1. In our model, the linear activation function is employed in the regression-based output layers to directly output unbounded numerical values, crucial for maintaining the scale of our target variable. Conversely, the softmax activation function, used in the classification layers, transforms raw neural network scores into probabilities, essential for distinguishing among categories like benign, malignant, or normal in mammogram imaging. While linear functions help preserve output consistency, softmax is vital for accurate multi-class classification, facilitating definitive diagnostic decisions.

## Results and discussion

### Mammogram images collection

Two major BC mammography image databases provided the input BC pictures needed to carry out the segmentation and detection functions in the implemented BC detection model. Table 2 contains information about the database and the sources from which the images are available. The term $B{C}_{fs}^{img}$ represents the collected images from the two standard databases.

**Table 2:** Description of a mammography image database.

S. No.	Database name	Image description	Available website
1	MIAS Mammography	Images and labels for mammography scans make up the database. This dataset consists of seven labels: reference number, background tissue’s characteristics, abnormality class, condition of abnormality, image coordinates, and radius of abnormality. It contains 1,679 images with labels. The images provided in this database are of size 1024 × 1024 pixels	https://www.kaggle.com/datasets/kmader/mias-mammography access date: 2023-01-11
2	CBIS-DDSM: Breast Cancer Image Dataset	There are 2620 digitized film mammography studies in the DDSM database. It includes instances with certified pathology data for normal, benign, and cancerous conditions. The DDSM is a helpful tool in designing and testing decision support systems due to the size of the database and ground truth checking. A trained radiologist chose and curated a subset of the DDDSM data for the CBIS-DDSM collection. After being decompressed, the images were changed to DICOM format. Together with pathologic diagnosis for training data, updated ROI segmentation and bounding boxes are also given	https://www.kaggle.com/datasets/awsaf49/cbis-ddsm-breast-cancer-image-dataset access date: 2023-03-07

### Experimental setup

The constructed DL-based BC detection model was assessed using the Python platform. The experimental results of this evaluation were further discussed. The DL-based BC detection model was constructed with an iteration count that should not exceed 50 and a maximum population size of 10, respectively. The MML-EOO-ACA-ATRUNet-AMDN-based BC detection framework was assessed against different classifiers like UNet^32^, KNN^29^, CNN^25^, XGBoost^24^, ResUNet+ + ^33^, GPA-TUNet^34^, and Deeplab^35^, and contrasted with existing meta-heuristic algorithms like Grey Wolf Optimization algorithm (GWO)-ACA-ATRUNet-AMDN^36^, Honey Badger Algorithm (HBA)-ACA-ATRUNet-AMDN^37^, JAYA-ACA-ATRUNet-AMDN^38^, and EOO-ACA-ATRUNet-AMDN^39^ algorithm for representing the accuracy of the developed deep learning-based BC detection model.

### Validation metrics used in evaluation

The below-provided metrics are utilized in assessing the implemented BC detection framework.

**Table:** 

$Np=\frac{\text{VW}}{TV+VW}\quad\quad (12)$	$pcn=\frac{TU}{TU+TV}\quad\quad (13)$	$Fd=\frac{\text{TV}}{TV+TU}\quad\quad (14)$
$Sp=\frac{\text{VW}}{VX+TU}\quad\quad (15)$	$Se=\frac{\text{TU}}{TU+VX}\quad\quad (16)$	$\text{Fs} = \frac{\text{2*TU}}{2*\left(TU+TV+VX\right)}\quad\quad (17)$
$Fn=\frac{\text{VX}}{TU+VX}\quad\quad (18)$	$Jd\left(M{I}_{ma}^{am},S{I}_{ad}^{TRU}\right)=\frac{\left|M{I}_{ma}^{am}\cap S{I}_{ad}^{TRU}\right|}{\left|M{I}_{ma}^{am}\cup S{I}_{ad}^{TRU}\right|}\quad\quad (19)$	
$$\text{Mc} = \frac{\text{TU*VW-TV*VX}}{\sqrt{\left(TU+TV\right)\left(TU+VX\right)\left(VW+TV\right)\left(VW+VX\right)}}\quad\quad (20)$$		

Negative Predictive Value (NPV) $Np$ is determined by Eq. (12). The precision ${\text{pcn}}$ is evaluated based on Eq. (13). False Discovery Rate (FDR) $Fd$ is computed as in Eq. (14). Specificity $Sp$ is determined as in Eq. (15). Matthews Correlation Co-efficient (MCC) $Mc$ is evaluated as provided in Eq. (20). Sensitivity $Se$ is calculated using Eq. (16). The F1 score ${\text{Fs}}$ is determined using Eq. (17). False Negative Rate (FNR) $Fn$ is evaluated using Eq. (18). The Jaccard distance $Jd$ between the ground truth image/mask images and the segmented image is computed using Eq. (19).

**Figure 3:**
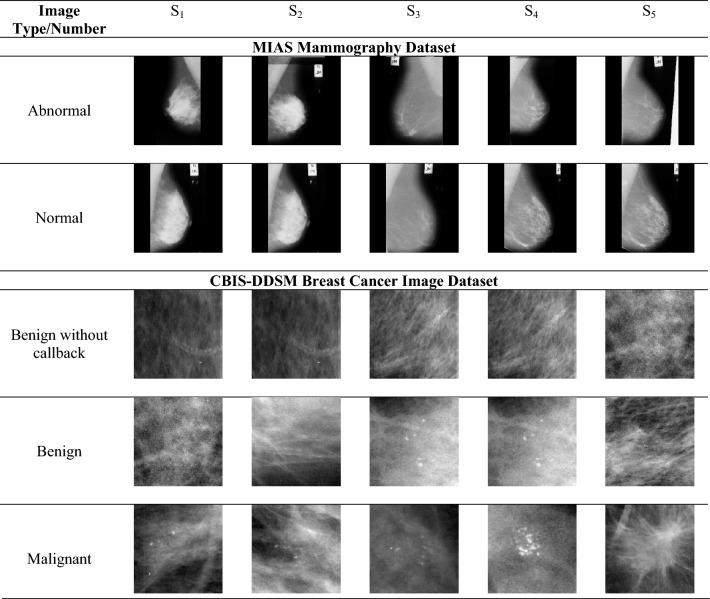
Sample Images(S_1_, S_2_, S_3_, S_4_, S_5_) Gathered from the MIAS and CBIS-DDSM Database.

The images gathered from the two databases are shown in Fig. 3.

### Experimental outcome

**Figure 4:**
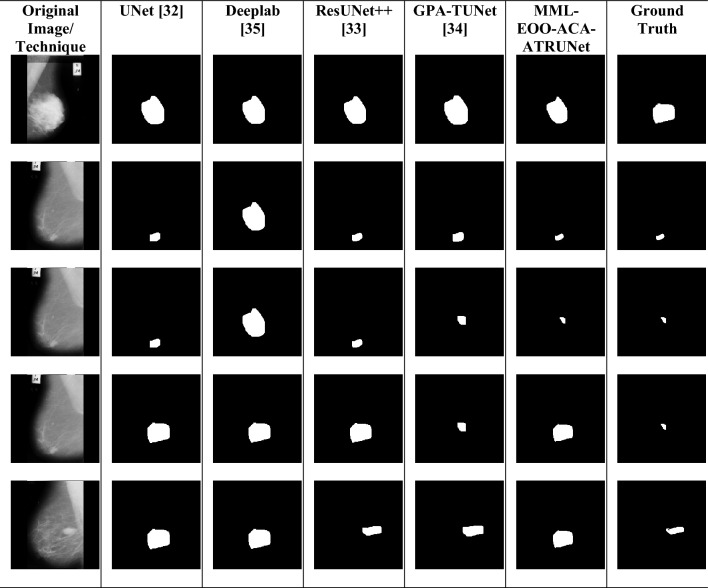
Segmented mammogram BC image outputs from proposed and conventional classifiers.

The segmented BC mammogram images obtained from various deep learning techniques and the ground truth comparison with the suggested MML-EOO-ACA-ATRUNet technique output are shown in Fig. 4.

### Performance comparison of the developed BC detection model with conventional classifiers

**Figure 5:**
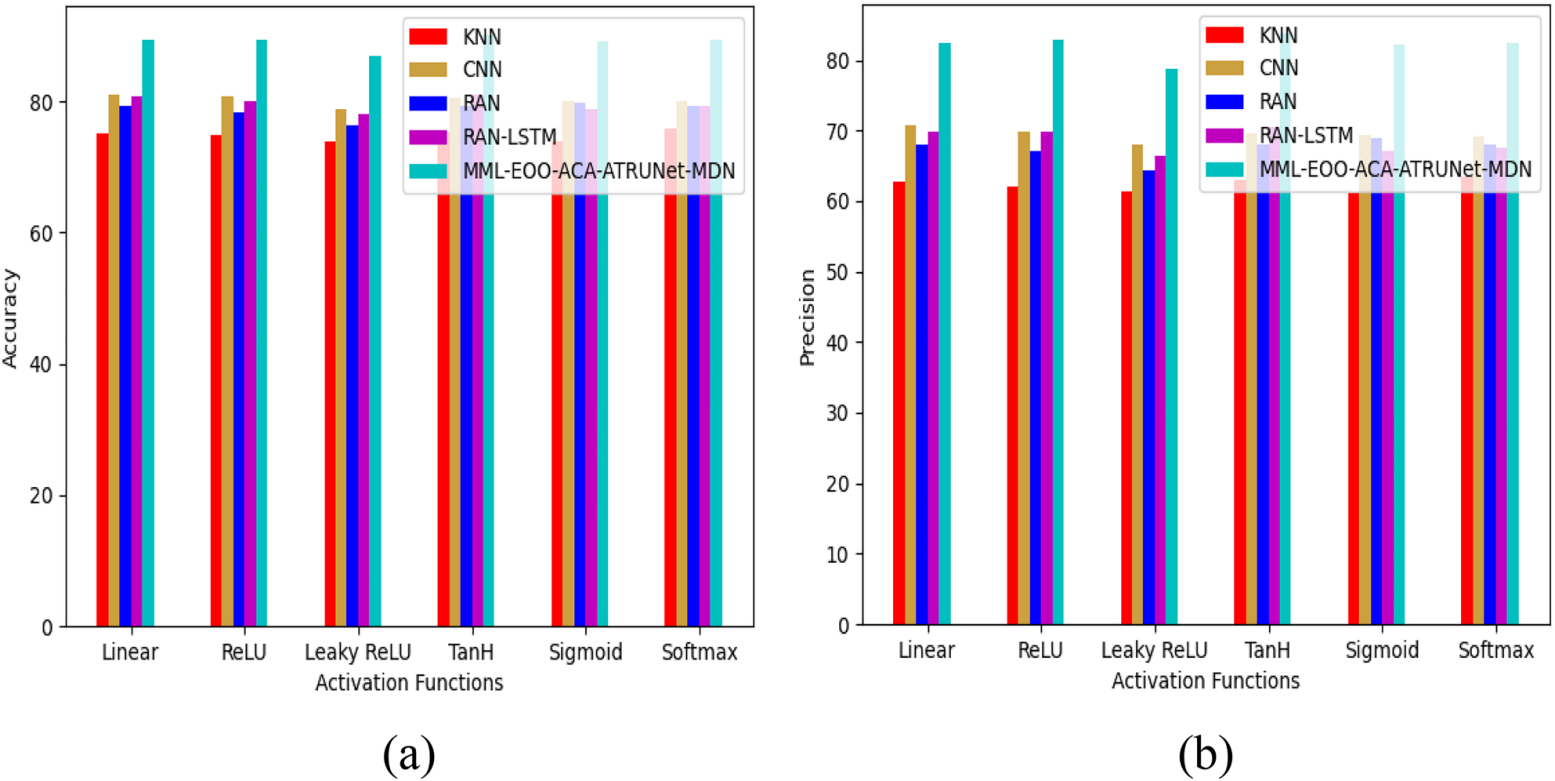
Performance comparison of the developed BC detection model with conventional classifiers with respect to MIAS Mammography Dataset in terms of “(**a**) accuracy, (**b**) precision.”

**Figure 6:**
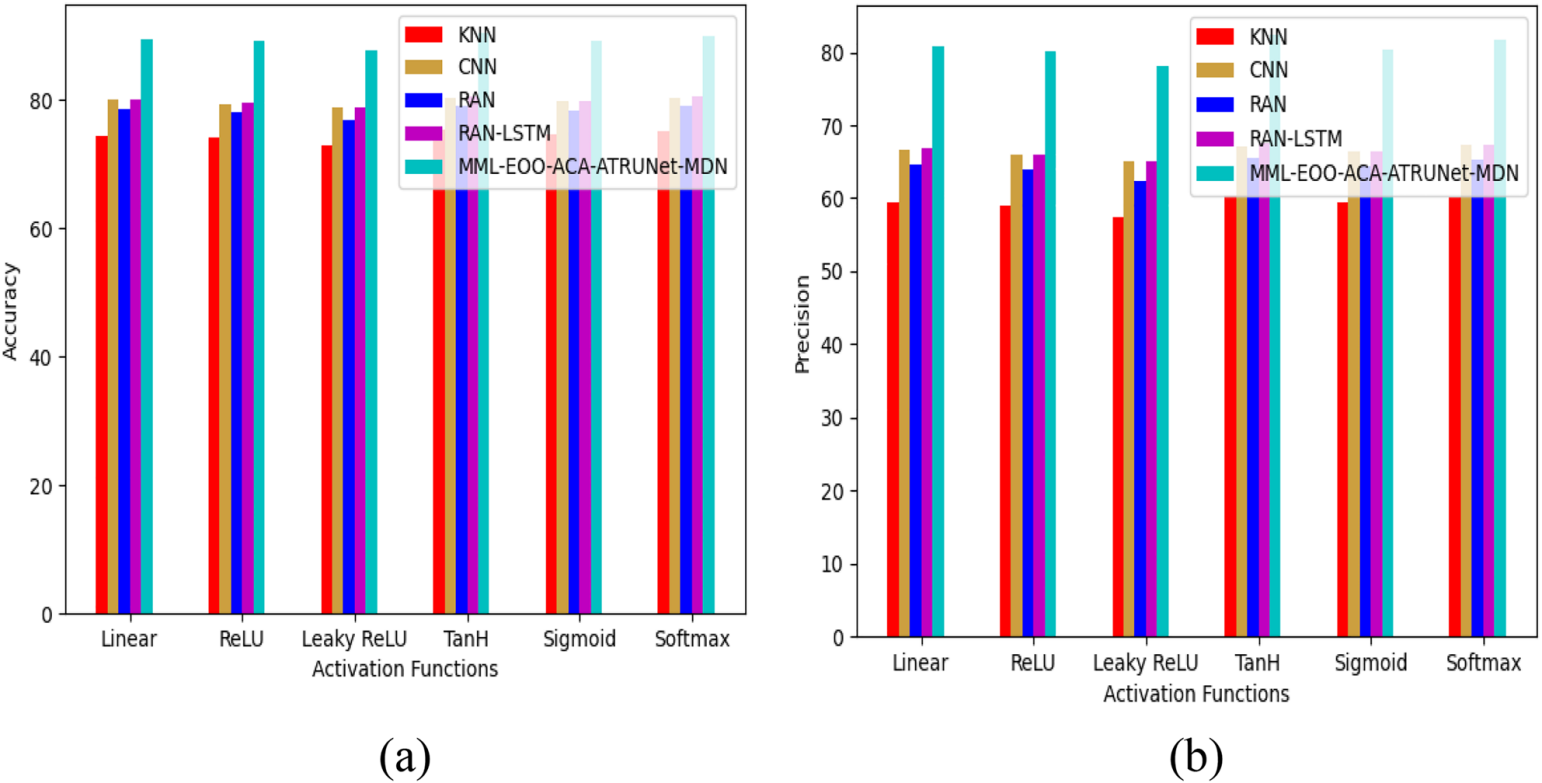
Performance comparison of the developed BC detection model with conventional classifiers with respect to the CBIS-DDSM breast cancer image dataset in terms of “(**a**) accuracy, (**b**) precision.”

The performance comparison of the developed MML-EOO-ACA-ATRUNet-MDN BC detection model with respect to conventional classifiers for the MIAS Mammography Dataset and CBIS-DDSM Breast Cancer Image Dataset is given in Figs. 5 and 6, respectively. The precision of the implemented MML-EOO-ACA-ATRUNet-MDN 5%, 2.56%, 3.8%, and 5.56% higher than the KNN, CNN, RAN, and RAN-LSTM classifiers for MIAS Mammography Dataset for ReLU activation function, respectively. We have mentioned the precision and accuracy results here; other evaluation measures' performance comparison results can be found in Appendix A: supplementary material.

### Comparison of the proposed BC detection framework with existing algorithms

**Figure 7:**
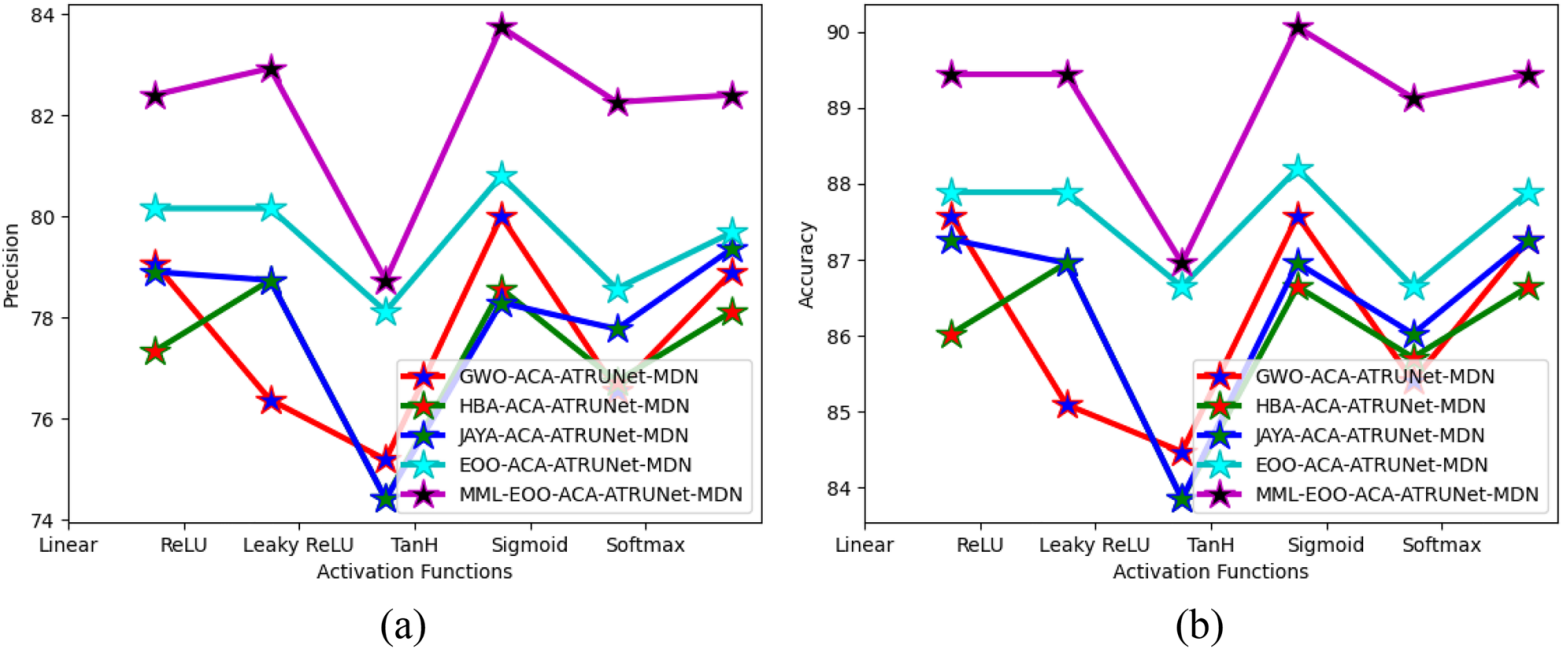
Evaluation of the recommended BC detection framework with existing algorithms with respect to the MIAS Mammography Dataset in terms of “(**a**) precision (**b**) accuracy.”

**Figure 8:**
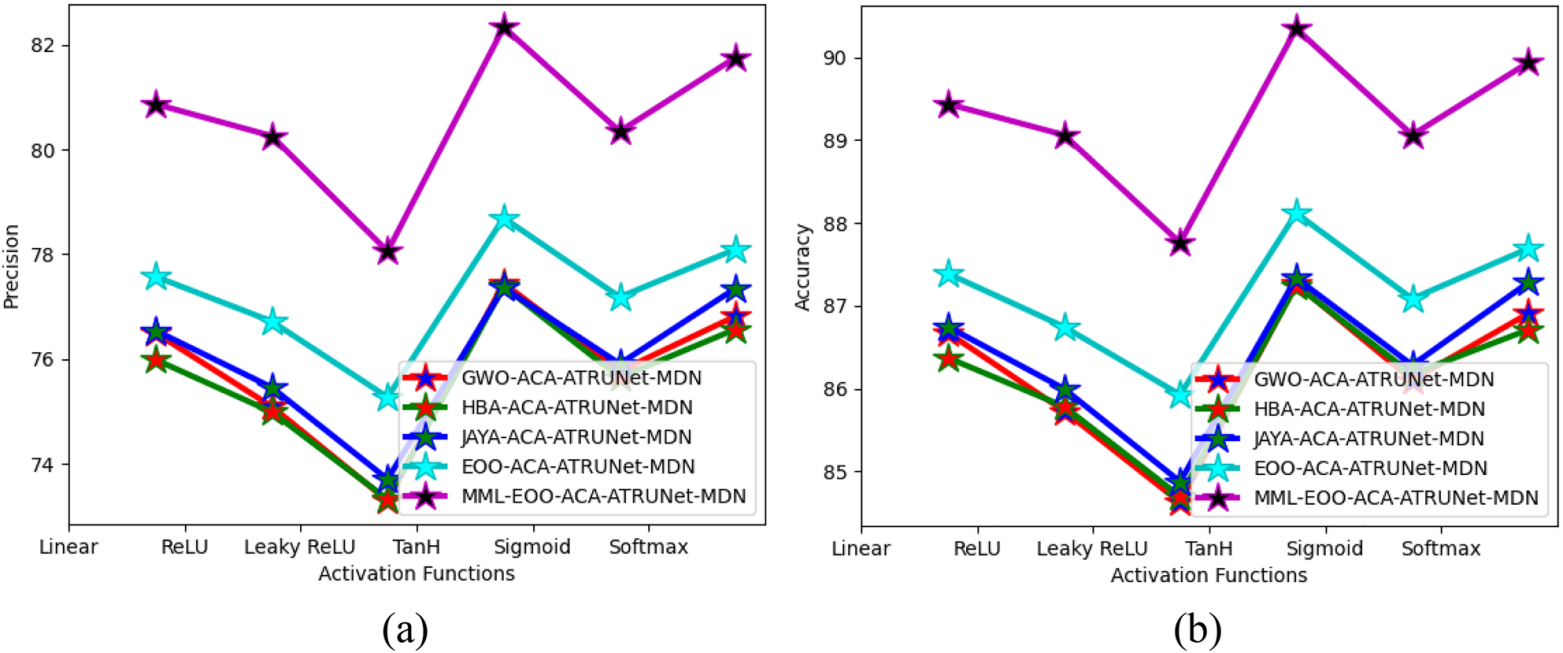
Evaluation of the recommended BC detection framework with existing algorithms with respect to CBIS-DDSM breast cancer image in terms of “(a) precision, (b) accuracy”.

The performance comparison of the proposed MML-EOO-ACA-ATRUNet-MDN BC detection model with respect to various existing algorithms for MIAS Mammography Dataset and CBIS-DDSM breast cancer image is given in Figs. 7 and 8, respectively. The accuracy of the proposed MML-EOO-ACA-ATRUNet-MDN-based BC detection framework is 2.32%, 3.27%, 3.39%, and 3.63% better than the EOO-ACA-ATRUNet-MDN, JAYA-ACA-ATRUNet-MDN, HBA-ACA-ATRUNet-MDN, and GWO-ACA-ATRUNet-MDN algorithms respectively for CBIS-DDSM breast cancer image on Leaky ReLU activation function.

### Statistical examination of the implemented BC detection framework with traditional classifiers

**Figure 9:**
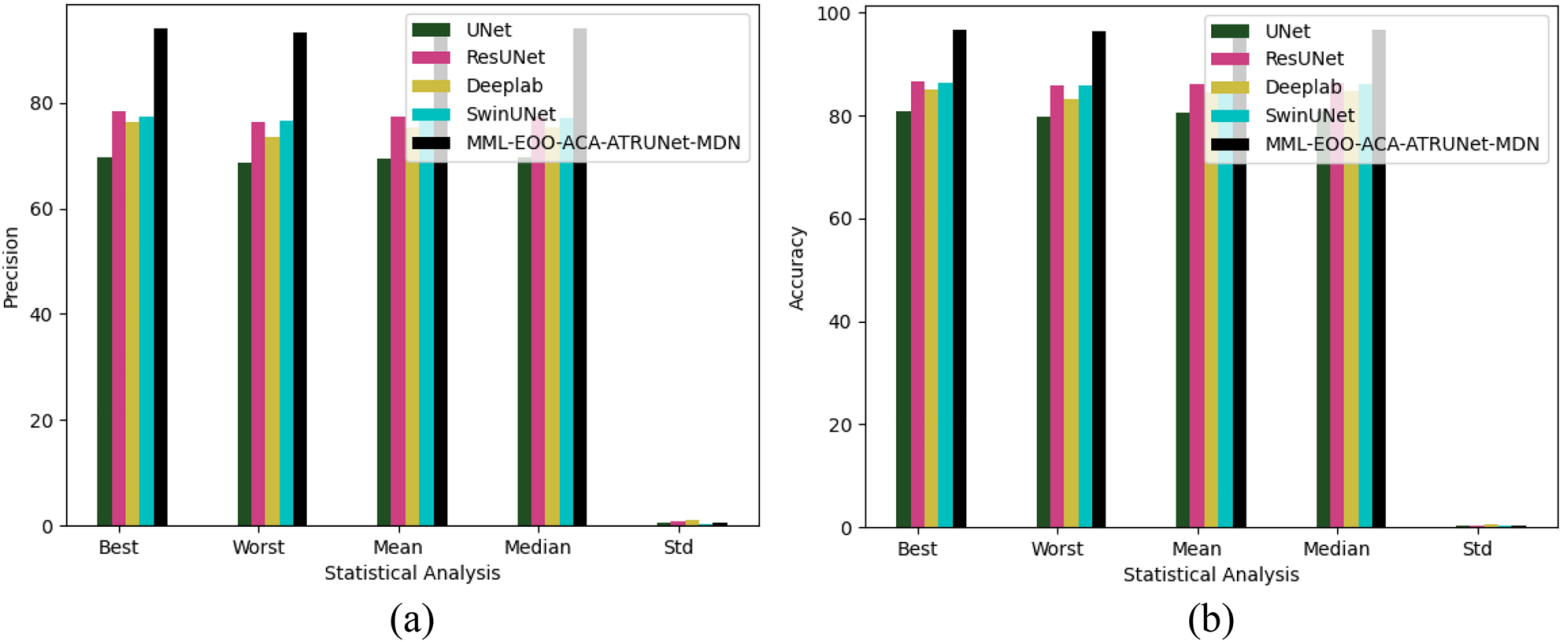
Statistical examination of the implemented BC detection framework with traditional classifiers with respect to MIAS Mammography Dataset in terms of “(**a**) precision, (**b**) accuracy.”

**Figure 10:**
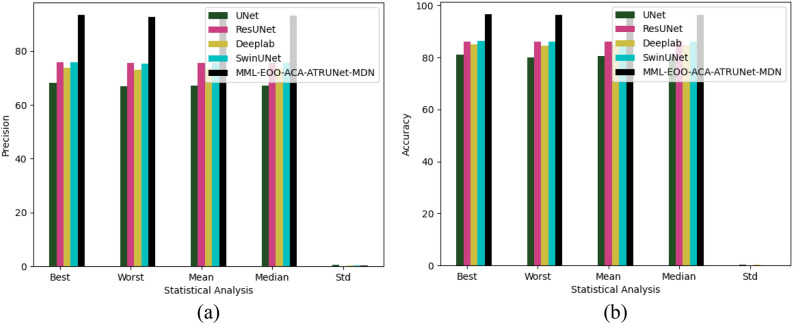
Statistical examination of the implemented BC detection framework with traditional classifiers with respect to CBIS-DDSM breast cancer image in terms of “(**a**) precision, (**b**) accuracy.”

The statistical examination of the implemented MML-EOO-ACA-ATRUNet-MDN-based BC detection framework to different traditional classifiers in the MIAS Mammography Dataset and CBIS-DDSM breast cancer image is shown in Figs. 9 and 10, respectively. The precision of the implemented MML-EOO-ACA-ATRUNet-MDN BC detection framework is 2.63%, 1.33%, 5.13%, and 5.71% better than the GPA-TUNet, Deeplab, ResUNet+ + , and UNet classifiers respectively for MIAS Mammography Dataset.

### Statistical assessment of the constructed BC detection model with other heuristic algorithms

**Figure 11:**
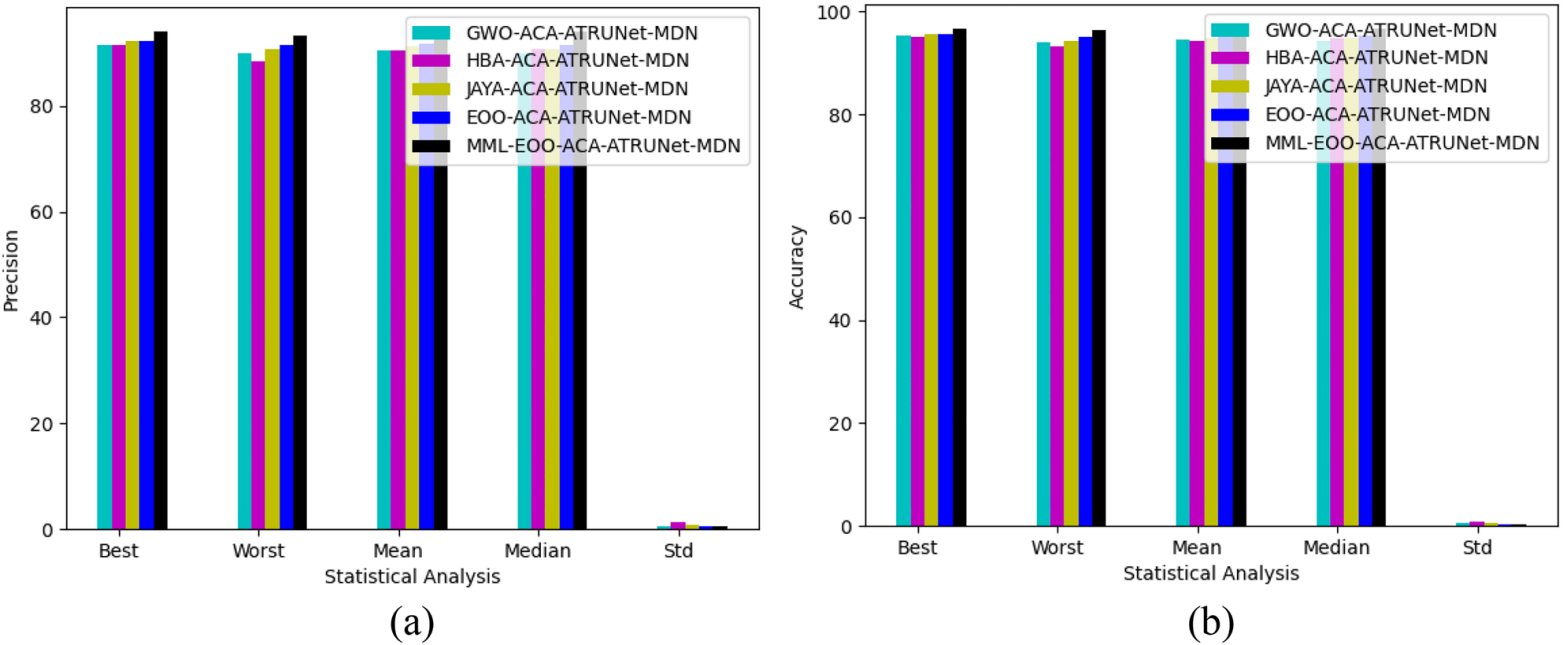
Statistical assessment of the constructed BC detection model with other heuristic algorithms with respect to MIAS Mammography Dataset in terms of “(**a**) precision, (**b**) accuracy.”

**Figure 12:**
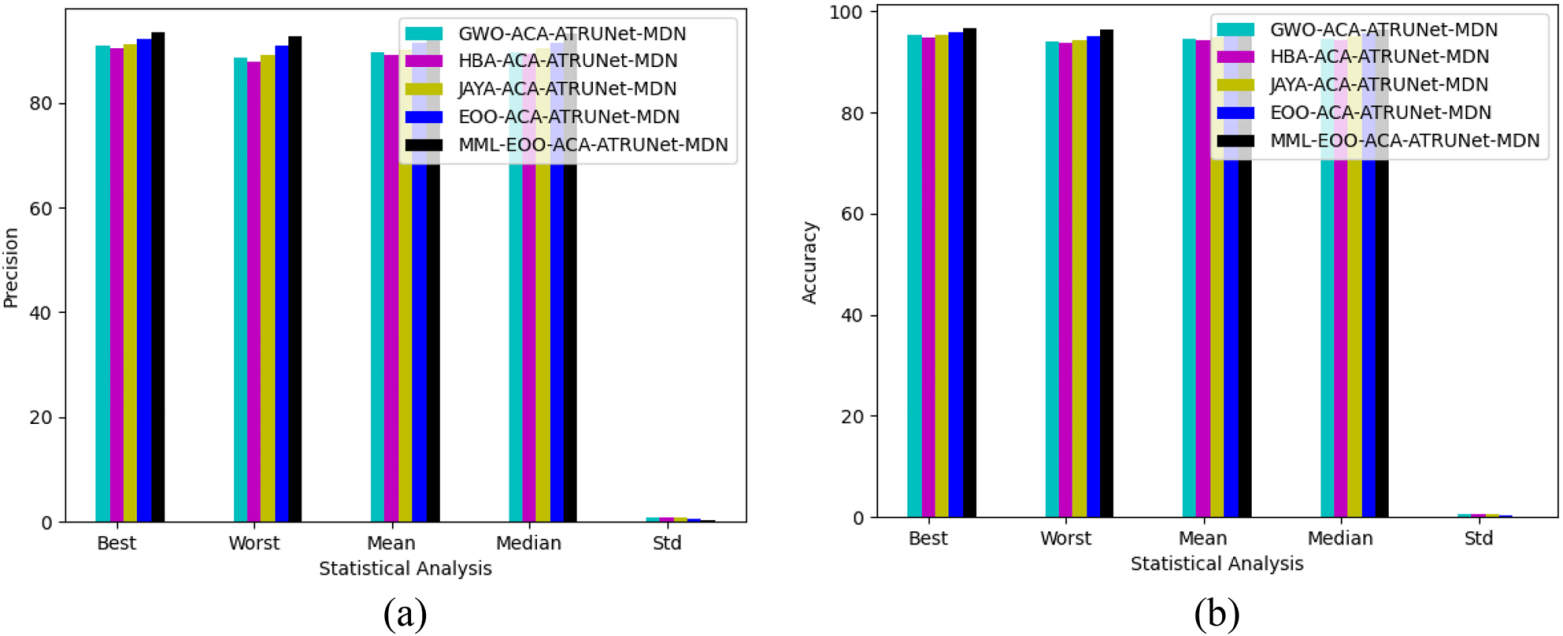
Statistical assessment of the constructed BC detection model with other heuristic algorithms with respect to CBIS-DDSM breast cancer image in terms of “(**a**) precision, (**b**) accuracy.”

The statistical assessment of the constructed MML-EOO-ACA-ATRUNet-MDN BC detection model to other heuristic algorithms in the MIAS Mammography Dataset and CBIS-DDSM breast cancer image is illustrated in Figs. 11 and 12, respectively. The sensitivity of the constructed MML-EOO-ACA-ATRUNet-MDN BC detection model is 1.04%, 2.11%, 2.65%, and 2.11% higher than the EOO-ACA-ATRUNet-MDN, JAYA-ACA-ATRUNet-MDN, HBA-ACA-ATRUNet-MDN, and GWO-ACA-ATRUNet-MDN algorithms respectively for MIAS Mammography Dataset.

### Cost function analysis of the generated BC detection framework

**Figure 13:**
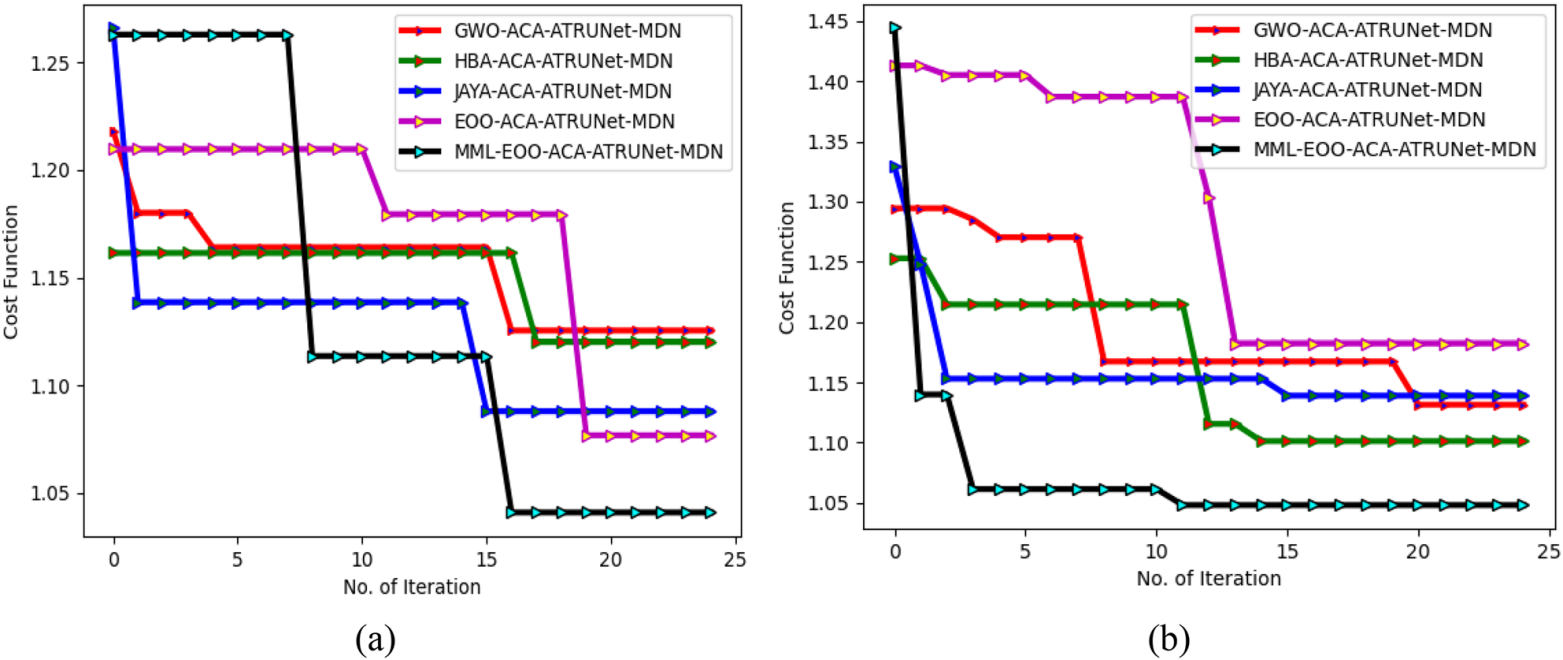
Cost function analysis of the generated BC detection framework in terms of “(**a**) MIAS Mammography Dataset, and (**b**) CBIS-DDSM breast cancer image.”

The cost function of the generated MML-EOO-ACA-ATRUNet-MDN-based BC detection framework is 7.87%, 9.4%, 13.11%, and 23.19% lesser than the JAYA-ACA-ATRUNet-MDN, GWO-ACA-ATRUNet-MDN, HBA-ACA-ATRUNet-MDN, and EOO-ACA-ATRUNet-MDN algorithms respectively for CBIS-DDSM breast cancer image shown in Fig. 13.

### Classifier analysis of the suggested MML-EOO-ACA-ATRUNet-MDN-based BC detection framework

The performance comparison of the suggested MML-EOO-ACA-ATRUNet-MDN-based BC detection framework is tabulated in Table 3. The precision of the suggested MML-EOO-ACA-ATRUNet-MDN-based BC detection framework is 34.52%, 18.41%, 19.32%, and 22.49% higher than the KNN, CNN, XGBoost, and ACA-ATRUNet-MDN classifiers respectively for MIAS Mammography Dataset.

**Table 3:** Comparison of the suggested BC detection framework with conventional classifiers. (Unit:%).

TERMS/Classifiers	KNN^29^	CNN^25^	XGBoost^24^	ACA-ATRUNet-MDN^40^	MML-EOO-ACA-ATRUNet-MDN
MIAS Mammography Dataset					
NPV	83.607	87.435	87.368	87.568	93.434
Accuracy	73.913	80.124	79.814	78.882	89.130
F1-Score	66.929	73.984	73.684	73.016	85.356
Specificity	73.913	80.676	80.193	78.261	89.372
Precision	61.151	69.466	68.939	67.153	82.258
FPR	26.087	19.324	19.807	21.739	10.628
MCC	0.463	0.583	0.578	0.565	0.769
Sensitivity	73.913	79.130	79.130	80.000	88.696
FDR	38.849	30.534	31.061	32.847	17.742
FNR	26.087	20.870	20.870	20.000	11.304
CBIS-DDSM Breast Cancer Image					
FPR	25.254	20.069	21.593	20.176	10.877
Sensitivity	74.185	79.583	78.087	79.904	88.936
Accuracy	74.559	79.815	78.300	79.850	89.061
NPV	85.274	88.675	87.739	88.820	94.156
Specificity	74.746	79.931	78.407	79.824	89.123
Precision	59.494	66.473	64.390	66.444	80.348
F1-Score	66.032	72.440	70.580	72.555	84.424
FNR	25.815	20.417	21.913	20.096	11.064
MCC	0.468	0.573	0.543	0.575	0.763
FDR	40.506	33.527	35.610	33.556	19.652

#### Algorithmic evaluation of the recommended MML-EOO-ACA-ATRUNet-MDN-based BC detection model

The algorithmic evaluation of the recommended MML-EOO-ACA-ATRUNet-MDN-based BC detection framework is tabulated in Table 4. The NPV of the recommended MML-EOO-ACA-ATRUNet-MDN-based BC detection model is 1.94%, 1.64%, 1.68%, and 1.2% better than the GWO-ACA-ATRUNet-MDN, HBA-ACA-ATRUNet-MDN, JAYA-ACA-ATRUNet-MDN, and EOO-ACA-ATRUNet-MDN algorithms respectively for CBIS-DDSM breast cancer image.

**Table 4:** Algorithmic evaluation of the recommended BC detection model. (Unit:%).

TERMS/Algorithm	GWO-ACA-ATRUNet-MDN^36^	HBA-ACA-ATRUNet-MDN^37^	JAYA-ACA-ATRUNet-MDN^38^	EOO-ACA-ATRUNet-MDN^39^	MML-EOO-ACA-ATRUNet-MDN
MIAS mammography dataset					
FPR	14.493	14.493	13.527	13.043	10.628
FDR	23.438	23.256	22.222	21.429	17.742
Sensitivity	85.217	86.087	85.217	86.087	88.696
NPV	91.237	91.710	91.327	91.837	93.434
Precision	76.563	76.744	77.778	78.571	82.258
Accuracy	85.404	85.714	86.025	86.646	89.130
FNR	14.783	13.913	14.783	13.913	11.304
Specificity	85.507	85.507	86.473	86.957	89.372
F1-Score	80.658	81.148	81.328	82.158	85.356
MCC	0.692	0.700	0.704	0.717	0.769
CBIS-DDSM breast cancer image					
Specificity	86.291	86.104	86.317	87.146	89.123
NPV	92.363	92.639	92.603	93.039	94.156
MCC	0.700	0.703	0.705	0.721	0.763
Precision	75.768	75.644	75.906	77.182	80.348
FPR	13.709	13.896	13.683	12.854	10.877
Accuracy	86.104	86.175	86.282	87.084	89.061
FDR	24.232	24.356	24.094	22.818	19.652
F1-Score	80.441	80.629	80.731	81.779	84.424
FNR	14.270	13.683	13.789	13.041	11.064
Sensitivity	85.730	86.317	86.211	86.959	88.936

## Conclusion

In this study, we have successfully developed and validated a highly accurate deep learning-based framework for breast cancer (BC) detection. The framework's robustness was first established through the collection of mammography images from benchmark datasets. A key innovation in our approach is the ACA-ATRUNet, a novel architecture combining Transformer blocks, ResNet, and UNet, which was meticulously tuned using the modified MML-EOO algorithm. This was crucial for effective segmentation, a foundational step in our two-phase detection process. The subsequent phase, actual BC detection, was executed using the ACA-AMDN, also fine-tuned with the MML-EOO algorithm. The combined use of these advanced technologies not only enhances the detection efficiency and precision but also addresses the common limitation in feature region size selection typically encountered in feature extraction networks.

## Data Availability

The datasets used/analyzed in our study are publicly available and were accessed as follows: The MIAS Mammography dataset, accessed on 2023-01-11, is available at https://www.kaggle.com/datasets/kmader/mias-mammography, the CBIS-DDSM: Breast Cancer Image Dataset, accessed on 2023-03-07, can be found at https://www.kaggle.com/datasets/awsaf49/cbis-ddsm-breast-cancer-image-dataset.
